# The psychosocial impact of a chronic disease in Ireland: Burdens and helpful practices for a life with epidermolysis bullosa

**DOI:** 10.1111/hex.14088

**Published:** 2024-06-21

**Authors:** Gudrun Salamon, Ursula Field‐Werners, Sophie Strobl, Vinzenz Hübl, Anja Diem

**Affiliations:** ^1^ Faculty of Psychology Sigmund Freud University Vienna Austria; ^2^ EB House Austria, Department of Dermatology and Allergology University Hospital of the Paracelsus Medical University Salzburg Austria

**Keywords:** burden, care practices, epidermolysis bullosa, healthcare system, health policy implications, quality of life, resources

## Abstract

**Objective:**

Although Ireland has one of the highest levels of well‐being in Europe, having a health condition has been found to have a direct negative impact. The aim of this study is to evaluate the current situation and the experiences of patients with epidermolysis bullosa (EB), a rare genetic skin disease, and their relatives living in Ireland, with a focus on burdens and helpful practices.

**Methods and Measures:**

In a mixed‐methods design, a series of standardised questionnaires were combined with open‐ended questions. Via an online survey, data from *n* = 59 EB patients and relatives of EB patients living in Ireland were collected.

**Results:**

EB affects both the patients and their relatives. Burdens were found in relation to the visibility of EB, the degree of severity, the current health status, reduced mobility, the financial impact of EB, the psychosocial impact and personal and social resources. The paper also analyses existing resources and highlights opportunities for support and needs of improvement.

**Conclusion:**

Quality of life with EB is influenced by somatic symptoms and the psychosocial burden. Individual helpful practices in dealing with this rare disease can be considered as mediators, but they need to be supported by structural and healthcare improvements.

**Patient or Public Contribution:**

The perspective of EB patients, their relatives and EB experts were taken into account in the development of the study design via two feedback loops with the EB patient organisations DEBRA Ireland and DEBRA Austria. The design was adapted accordingly. Additionally, by including open‐ended questions, patients and relatives could contribute their individual perspectives and add insights into their lives with EB that might not have been captured with the structured online survey alone.

## 1 INTRODUCTION

Epidermolysis bullosa (EB) is a genetic disease affecting the skin. Even minimal trauma and friction can lead to painful wounds and blister formation. The clinical picture of EB varies individually and is classified into four main types EB simplex (EBS), dystrophic EB (DEB), junctional EB (JEB) and Kindler EB (KEB), which are again differentiated into nearly 30 subtypes.^1^ With no cure in sight yet, patients and their families need to deal with the pain, the wound care and other effects of EB on a daily basis. Daily wound care and dressing changes are highly painful, need special bandages, creams and ointments and may take two to 3 h/day.^2^, ^3^, ^4^ As EB is a multisystemic condition, people affected are restricted in their daily activities, which leads to a reduced quality of life.^5^, ^6^, ^7^, ^8^ Furthermore, EB comes with a variety of psychosocial effects, ranging from economic difficulties and social isolation to mental health problems such as constant worry, depression or anxiety.^9^, ^10^, ^11^, ^12^, ^13^ However, EB affects not only the patients themselves but can also put tremendous stress on the whole family. Relatives often have to take on the role of caregiver, provide emotional and social support and manage organisational and economic obstacles. EB therefore often determines many aspects of their lives, posing an emotional, social and economic challenge.^14^, ^15^, ^16^, ^17^

Worldwide, approximately 500,000 individuals are affected by this rare disorder. Regarding the prevalence of EB in Ireland, one in 18,000 children is born with EB and around 200–300 families are affected by EB.^18^, ^19^, ^20^ According to the European Social Survey, Ireland has one of the highest levels of well‐being in Europe among 26 countries. Yet, people with a health condition had a significant decrease in their well‐being.^21^ There is still a serious demand for research into the needs of Irish patients and their families, especially in the case of rare diseases.^22^ So far, previous EB studies conducted in Ireland primarily focussed on establishing, maintaining and improving a supportive health care system* and the quality of care.^19^, ^23^, ^24^ However, the psychosocial intra‐ and interindividual impact of EB on affected individuals and their families has not been adequately studied with a focus on the Irish population.

The aim of the current study is to analyse public health practices in Ireland and to identify the burdens of Irish EB patients and their families and helpful practices and resources in managing this disease.

## 2 MATERIALS AND METHODS

### 2.1 Recruitment and design

The study participants for this online survey were recruited in collaboration with the patient organisation DEBRA Ireland and DEBRA International. The study inclusion criteria were a diagnosis of EB or being a relative of a person living with EB, a minimum age of 14 years and fluency in English. Informed consent or assent was obtained from all participating patients or legal guardians of underage patients, respectively. The study design builds upon a previous qualitative study by the same research group on life with EB. In two feedback loops with the EB patient organisations DEBRA Ireland and DEBRA Austria, patients', relatives' and experts' experiences were included in the study and the design adapted accordingly.

The survey took place between April and September 2021 and consisted of sociodemographic questions, a series of standardised questionnaires and additional open‐ended questions. The current health status was assessed by the Instrument for Scoring Clinical Outcomes of Research for Epidermolysis Bullosa (iscorEB).^25^, ^26^, ^27^ The quality of life of the patients was evaluated by the Quality of Life in Epidermolysis Bullosa (QOLEB)^28^, ^29^ and of the family members by the Epidermolysis Bullosa Burden of Disease (EB‐BoD).^30^ The Resources for Life with an Illness for Epidermolysis Bullosa (ResILL‐EB) questionnaire assesses burdens, satisfaction, resources and helpful practices.^31^ Furthermore, three questionnaires for the elicitation of different aspects of resilience were used, namely the Satisfaction with Life Scale (SWL‐5),^32^, ^33^ the Perceived Social Support Questionnaire (F‐SozU)^34^, ^35^ and the Brief Resilience Scale (BRS).^36^, ^37^ Participants also completed open‐ended questions regarding further burdens and helpful practices, additional support and their stress management. With the exception of the sociodemographic questions on EB and the current state of health, the relatives were asked to answer all questions referring to their own situation. To take into account the unpredictability of EB and to reduce drop‐outs for health reasons, the option was offered to pause the questionnaire for a maximum of 7 days and continue at a later date.

### 2.2 Data analysis

As a first step, the requirements for the appropriate statistical tests were investigated, the dependent variables were inspected by the use of histograms, scatterplots and Kolmogorov–Smirnov tests, skewness and kurtosis statistics were computed. To describe the sample, crosstabs, frequencies, percentages, means, medians, interquartile range and standard deviations were calculated. Due to the open online survey and the recruitment via stakeholders, no exact participation ratio can be calculated. To evaluate the relation between dichotomous and continuous variables, point‐biserial correlations were calculated. For group comparisons, nonparametric *U*‐tests and Kruskal–Wallis tests were applied. To counterbalance missing data and include as much collected data as possible, calculations for questionnaires were based on average scores instead of sum scores. A significance level of 5% was assumed for all statistical calculations. The statistical software program SPSS, version 27, was used to analyse the collected data.

Concerning the open‐ended questions, answers were coded and analysed on the basis of a structured coding system, in accordance with the recommendations given by the Reflexive Thematic Analysis methodology.^38^, ^39^ Coding was carried out using MAXQDA software, version 2022, enabling automated quantification of code occurrence. Topics frequently mentioned in the qualitative data were further investigated by explorative quantitative comparison of content‐related single items of a scale concerning the given topic (e.g., item ‘mood’ of the iscorEB).

## 3 RESULTS

### 3.1 Sample characteristics

A total of 59 participants, 28 EB patients and 31 family members of EB patients took part in the online survey. Regarding the gender of the participants, the vast majority were women (76.3%). The average age of the patients was 38.89 (±12.23) years, while the mean of the family members was 47.55 (±12.99) years. The average age of their relatives' person with EB was 18.32 (±18.95). The majority of the participants lived in or close to a city (59.6%) and were native English speakers (78.0%), the remaining participants stated that their level of English was very good to good.

Regarding the different types of EB, almost two‐thirds of all participants were either diagnosed with or had a relative with EBS (64.4%), followed by DEB (30.5%, whereof 15.3% had RDEB and 13.6% had DDEB) and JEB (5.1%). The degree of severity (mild: 40.7%; moderate: 37.3%; severe: 22.0%; see Table A1) and visibility (not: 16.9%; rather not: 20.3%; somewhat: 35.6%; very: 27.1%) was well distributed. At the time point of the survey, 39.3% of participants indicated an acute challenging phase with regard to EB, and three stated that they or their relatives were in the palliative phase (5.1%). With regard to the most commonly affected body parts, the vast majority reported EB symptoms on the feet (85.7%) and hands (75.0%), followed by the legs (39.3%) and the arms (25.0%). A high percentage of the participants stated that the mobility was reduced due to the legs/feet (61.0%) and/or hands (30.5%). In total, 8.5% of the EB patients used a walking aid or wheelchair and 3.4% a grip aid. Overall, the rate of missing data in the survey was 3.4%. An overview of the sample characteristics can be found in Table 1.

**Table 1:** Sociodemographic characteristics of the patients and relatives.

		Patients		Relatives		All		
Sample size		28		31		59		
Average age		38.89 (SD = 12.23; MD = 39.00)		47.55 (SD = 12.99; MD = 48.00)		43.44 (SD = 13.26; MD = 42.00)		
		Range from 17 to 66 years		Range from 21 to 76 years		Range from 17 to 76 years		
		Participants (*n*)	Percent	Participants (*n*)	Percent	Participants (*n*)	Percent	
Gender	Female	20	71.4	25	80.6	45	76.3	
	Male	8	28.6	6	19.4	14	23.7	
	Residence						
Residence	In a city	13	46.6	6	19.4	19	32.3	
	Close to a city	7	25.0	15	48.4	22	37.3	
	Far away from a city	8	28.6	10	32.3	18	30.5	
Language level	Native	22	78.6	24	77.4	46	78.0	
	Very well	4	14.3	7	22.6	11	18.6	
	Well	2	7.1	0	0	2	3.4	
Relationship	Yes	21	75.0	24	77.4	45	76.3	
	No	7	25.0	7	22.6	14	23.7	
Relation to EB	Patients	28	100	‐		28	47.5	
	Parent/step‐parent/foster parent of a person with EB			25	80.65	25	42.4	
	Partner of a person with EB			1	3.23	1	1.7	
	Grandmother/grandfather of a person with EB			1	3.23	1	1.7	
	Aunt/uncle of a person with EB			1	3.23	1	1.7	
	Child/step‐child/foster child of a person with EB			3	9.67	3	5.1	
				Concerning their relative with EB		
Average age of the relative with EB				18.32 (SD = 18.95; MD = 13.00) Range from 1 to 73 years		
EB type	EBS	27	96.5	11	35.5	38	64.4	
	JEB	0	0	3	9.7	3	5.1	
	DEB	1	3.6	17	54.8	18	30.5	
	KEB	0	0	0	0	0	0	
EB severity	Mild	16	96.4	8	25.8	24	40.7	
	Moderate	12	3.6	10	32.3	22	37.3	
	Severe	0	0	13	41.9	12	22.0	
EB visibility	Not	5	17.9	5	16.1	10	16.9	
	Rather not	11	39.3	1	3.2	12	20.3	
	Somewhat	10	35.7	11	35.5	21	35.6	
	Very	2	7.1	14	45.2	16	27.1	
Last challenging phase in regard to EB	At the moment	11	39.3	5	16.1%	16	27.1	
	<6 months ago	16	57.1	12	38.7	28	47.4	
	6–12 months ago	0	0%	0	0	0	0	
	>12 months ago	1	3.6%	14	45.2	15	20.3	
Palliative phase	Yes	2	7.1	1	3.2	3	5.1	
	No	25	89.3	29	93.5	54	91.5	
	Missing	1	3.6	1	3.2	2	3.4	
Average time spent a week on EB‐specific things in hours		MD = 1 h (IQR = 1;3)		MD = 10 h (IQR = 3.5;20.5)		MD = 3.5 h (IQR = 1;10.88)		

Abbreviations: EB, epidermolysis bullosa; EBS, EB simplex; DEB, dystrophic EB; IQR, interquartile range; JEB, junctional EB; KEB, kindler EB; MD, mean deviation; SD, standard deviation.

### 3.2 Aspects influencing life with EB

To capture the full diversity of living conditions with EB, the subsequent calculations were performed for the subgroups of patients and relatives separately and thereafter for the combined data set. To be able to cover the broad spectrum of psychosocial effects of EB in this paper, only significant results are presented. Table 2 provides a correlation matrix between the questionnaires. A group comparison matrix of all categories' medians can be found in the additional material (Table A2).

**Table 2:** Cross‐correlation matrix of the questionnaires.

		Quality of life ↓		EB Burden ↑	Satisfaction ↑	Resources ↑	Helpful practices ↑	Resilience ↑	Social Support ↑	Overall satisfaction ↑
	Assessed by	For patients: QOLEB	for relatives: EB‐BoD	ResILL‐EB Burden	ResILL‐EB Satisfaction	ResILL‐EB Resources	ResILL‐EB Helpful practices	BRS	F‐SozU	SWLS
**Current health status ↓**	**iscorEB**	*ρ* = 0.595	*ρ* = 0.790	*ρ* = 0.676	*ρ* = −0.365	*ρ* = −0.203	*ρ* = −0.024	*ρ* = −0.006	*ρ* = −0.110	*ρ* = −0.372
		*p* < .001	*p* < .001	*p* < .001	*p* = .007	*p* = .142	*p* = .867	*p* = .966	*p* = .451	*p* = .009
**Quality of life ↓**	**For patients: QOLEB**	—	—	*ρ* = 0.712	*ρ* = −0.597	*ρ* = −0.405	*ρ* = −0.183	*ρ* = −0.357	*ρ* = −0.387	*ρ* = −0.581
				*p* < .001	*p* = .001	*p* = .036	*p* = .383	*p* = .080	*p* = .056	*p* = .002
	**For relatives: EB‐BoD**	—	—	*ρ* = 0.856	*ρ* = −0.270	*ρ* = −0.517	*ρ* = −0.001	*ρ* = −0.488	*ρ* = −0.277	*ρ* = −0.475
				*p* < .001	*p* = .191	*p* = .008	*p* = .996	*p* = .018	*p* = .200	*p* = .022
**EB Burden ↑**	**ResILL‐EB Burden**	*ρ* = 0.712	*ρ* = 0.856	—	*ρ* = −0.267	*ρ* = −0.460	*ρ* = −0.051	*ρ* = −0.250	*ρ* = −0.275	*ρ* = −0.374
		*p* < .001	*p* < .001		*p* = .049	*p* < .001	*p* = .717	*p* = .080	*p* = .053	*p* = .007
**Satisfaction ↑**	**ResILL‐EB Satisfaction**	*ρ* = −0.597	*ρ* = −0.270	*ρ* = −0.267	—	*ρ* = 0.189	*ρ* = −0.101	*ρ* = 0.291	*ρ* = 0.315	*ρ* = 0.584
		*p* < .001	*p* = .191	*p* = .049		*p* = .166	*p* = .475	*p* = .040	*p* = .026	*p* < .001
**Resources ↑**	**ResILL‐EB Resources**	*ρ* = −0.405	*ρ* = −0.517	*ρ* = −0.460	*ρ* = 0.189	—	*ρ* = 0.440	*ρ* = 0.564	*ρ* = 0.390	*ρ* = 0.461
		*p* = .036	*p* = .008	*p* < .001	*p* = .166		*p* = .001	*p* < .001	*p* = .005	*p* < .001
**Helpful practices ↑**	**ResILL‐EB Helpful practices**	*ρ* = −0.183	*ρ* = −0.001	*ρ* = −0.051	*ρ* = −0.101	*ρ* = 0.440	—	*ρ* = 0.091	*ρ* = 0.485	*ρ* = 0.214
		*p* = .383	*p* = .996	*p* = .717	*p* = .475	*p* = .001		*p* = .530	*p* < .001	*p* = .136
**Resilience ↑**	**BRS**	*ρ* = −0.357	*ρ* = −0.488	*ρ* = −0.250	*ρ* = 0.291	*ρ* = 0.564	*ρ* = 0.091	—	*ρ* = 0.326	*ρ* = 0.342
		*p* = .080	*p* = .018	*p* = .080	*p* = .040	*p* < .001	*p* = .530		*p* = .021	*p* = .015
**Social support ↑**	**F‐SoZU**	*ρ* = −0.387	*ρ* = −0.277	*ρ* = −0.275	*ρ* = 0.315	*ρ* = 0.390	*ρ* = 0.485	*ρ* = 0.326	—	*ρ* = 0.468
		*p* = .056	*p* = .200	*p* = .053	*p* = .026	*p* = .005	*p* < .001	*p* = .021		*p* < .001
**Overall satisfaction ↑**	**SWLS**	*ρ* = −0.581	*ρ* = −0.475	*ρ* = −0.374	*ρ* = 0.584	*ρ* = 0.461	*ρ* = 0.214	*ρ* = 0.342	*ρ* = 0.468	—
		*p* = .002	*p* = .022	*p* = .007	*p* < .001	*p* < .001	*p* = .136	*p* = .015	*p* < .001	

*Note*: ↑ = Higher values indicate higher expression in the targeted construct; ↓ = Lower values indicate higher expression in the targeted construct.

Abbreviations: BRS, Brief Resilience Scale; EB‐BoD, Epidermolysis Bullosa Burden of Disease; F‐SozU, Perceived Social Support Questionnaire; iscorEB, Instrument for Scoring Clinical Outcomes of Research for Epidermolysis Bullosa; QOLEB, Quality of Life in Epidermolysis Bullosa; ResILL‐EB, Resources for Life with an Illness for Epidermolysis Bullosa; SWL, Satisfaction with Life Scale.

### 3.3 Patients

Regarding the degree of severity, patients who rated their EB as mild had significantly more resources available than those with a moderate degree of severity (*U* = 44.50, z = −2.23, *p* = .026).

Furthermore, patients who stated that they were in an acute challenging phase reported significantly more EB symptoms as assessed by the iscorEB (*U* = 155.50, *z* = 2.92, *p* = .003), were more burdened (ResILL‐EB Burden scale: *U* = 130.00, *z* = 2.07, *p* = .038) and rated their quality of life lower (QOLEB: *U* = 143.00, *z* = 2.34, *p* = .019).

### 3.4 Relatives

In contrast to the patients subgroup, relatives reported that their person with EB had a higher degree of severity and more medical symptoms as assessed via iscorEB. With a high degree of severity, relatives reported to have a higher burden and a lower quality of life. There were significant differences between mild and severe EB cases with regard to the ResILL‐EB Burden scale (*H*(2) = 11.09, *p* = .004) and their quality of life of the relatives differed significantly between all three severity cases (EB‐BoD: *H*(2) = 9.95, *p* = .007). Similarly, with a high reduction in mobility of the person with EB, relatives reported a significantly lower quality of life (*U* = 138.00, *z* = 2.78, *p* = .005).

Moreover, women reported more burdens and had a lower quality of life than men. Significant differences between female and male participants were found in the ResILL‐EB Burden scale (*U* = 27.00, *z* = −2.19, *p* = .029) and the EB‐BoD (*U* = 14.00, *z* = 2.51, *p* = .012).

### 3.5 All participants

With regard to all participants, the results show that gender has an important influence on the perceived ***overall burden***, as measured by the ResILL‐EB Burden scale. Female participants indicated a significantly higher burden than male (*U* = 182.00, *z* = −2.03, *p* = .042). Patients and relatives who reported reduced mobility due to affected legs/feet and/or hands were more burdened than those who were not restricted (*U* = 468.50, *t* = 3.49, *p* < .001), and the same applies to participants who rated their or their relative's EB as very visible (*H*(3) = 8.02, *p* = .046). In addition, Table 2 shows that a higher level of stress correlates significantly with a poorer health status, a lower quality of life, less satisfaction and fewer resources.

Patients and relatives reporting a higher ***degree of severity*** tended to report more EB symptoms, more burdens associated with EB and fewer resources to deal with the disease. Significant differences were found on the iscorEB (*H*(2) = 15.75, *p* < .001), the ResILL‐EB Burden scale (*H*(2) = 16.73, *p* < .001) and the ResILL‐EB Resources scale (*H*(2) = 6.27, *p* = .044).

Regarding the ***current health status*** as assessed by the iscorEB, several significant group differences were found. EB patients who were in an acute challenging phase at the time point of the survey scored significantly higher on the iscorEB scale than those who were not (*U* = 452.50, *z* = 2.21, *p* = .027). Moreover, participants who stated that their or their relative's EB was not or hardly visible reported fewer EB symptoms than those with somewhat to very visible EB (*U* = 508.50, *z* = 2.03, *p* = .043).

The qualitative data underline the emotional impact regarding the ***visibility*** of EB for patients and for their families. The unpleasant reactions from the social environment as well as, for parents, the fear of being perceived as a bad parent evoke feelings of shame and are described as especially burdening.

The stares from other people, getting it into your own head that these people think that they were harmed by you or why they have these wounds. (1922, Pos. 2)

The ignorance of some people, looking at me thinking I am not a good mother because of how my darling girl looks. Though it breaks my heart, but I know she is all I will ever need/want in my life. (2543, Pos. 2)

However, not having visible wounds and blisters is also perceived as burdensome:

No‐one outside the family understands their pain as theirs is a hidden disability. (2306, Pos. 3)

***Reduced mobility*** because of EB also has an impact on the quality of life. Patients with restricted mobility had significantly more medical symptoms than those who were not limited (iscorEB‐p: *U* = 263.50, *z* = −1.99, *p* = .046) and they and their relatives were less satisfied with life (ResILL‐EB Satisfaction scale: *U* = 513.50, *z* = 2.59, *p* = .010; SWLS scale: *U* = 468.50, *z* = 3.49, *p* < .001). Patients whose legs/feet and hands were affected by EB and their relatives scored significantly lower on the resources scale than those who reported none or hardly any mobility restrictions (*U* = 503.50, *z* = 2.42, *p* = .015).

With reduced mobility, walking distances must be carefully evaluated and, depending on respective distances, affected individuals cannot participate in social activities. The constant necessity of planning ahead can be seen as an additional burden in itself. The struggles accompanying reduced mobility influence almost every aspect of daily life, e.g. grocery shopping, and can compromise the participants' autonomy and job opportunities.

How to avoid getting blisters on hands and feet for me and my kids. We would all love to travel (walk) and play sport, especially during the summer. (1869, Pos. 2)

I have to drive everywhere as walking to/from public transport is not an option, but finding parking is a real difficulty at times and I avoid many places, events and social outings as a result. (2562, Pos. 3)

We cannot do ‘normal’ things without extreme planning. Even doing my weekly grocery shopping can be hard if I have to park a long way from the door. (2198, Pos. 5)

The choice of professions are restricted to non‐physical careers which is extremely frustrating as it rules out so many opportunities. (2303, Pos. 3)

Participants with a high ***financial burden*** reported more medical symptoms as assessed by the iscorEB (iscorEB: *U* = 549.50, *z* = 3.22, *p* = .001) and were less satisfied with their situation (ResILL‐EB Satisfaction scale: *U* = 250.00, *z* = −2.16, *p* = .031; SWLS: *U* = 188.00, *z* = −2.42, *p* = .015). Regarding the items of the ResILL‐EB Burden und Satisfaction scales ‘I'm burdened by/satisfied with my financial situation’, only 22% of the participants stated that they were not burdened at all by EB, and only 8.5% were very satisfied with their financial situation.

Financial aspects emerged as one of the most prominent themes from the qualitative data. The economic costs are linked to a variety of outlays, ranging from affording proper clothes/footwear to medical and psychological treatment and even leisure activities, as shown in the statements below:

Financial support if you can't work from the government better than it is. (2417, Pos. 5)

Afford good footwear. (2788, Pos. 5)

Wound care products/bandages more readily available and at an affordable cost. (2789, Pos. 5)

There is a feeling of guilt if we go swimming and we have to change the dressings more often as I know how much they cost. (2423, Pos. 2)

1:1 psychotherapy should be freely available and linked to the treating team. (2831, Pos. 3)

The ***psychosocial impact*** of EB, as described by the participants, covers a variety of factors that lead to intrapersonal stress. With regard to the ‘usual mood’ as assessed via iscorEB, participants who stated that they were unhappy were more burdened, less satisfied with life, had fewer resources available, were less resilient and rated their quality of life lower. Significant differences were found on the ResILL‐EB Burden scale (*U* = 467.50, *z* = 3.10, *p* = .002), the ResILL‐EB Satisfaction scale (*U* = 184.00, *z* = −2.27, *p* = .023), the SWLS (*U* = 108.50, *z* = −3.19, *p* = .001), the ResILL‐EB Resources scale (*U* = 96.00, *z* = −3.95, *p* < .001), the BRS (*U* = 131.50, *z* = −2.69, *p* = .007), the QOLEB for the patients (*U* = 137.00, *z* = 3.38, *p* = .001) and on the EB‐BoD for the relatives (*U* = 121.50, *z* = 2.43, *p* = .015). With regard to the burden concerning ‘my feelings’ and ‘my worries and fears’ as assessed via ResILL‐EB Burden scale, participants who scored higher reported significantly more medical symptoms (‘my feelings’: *U* = 594.00, *z* = 4.16, *p* < .001; ‘my worries and fears’: *U* = 586.00, *z* = 3.88, *p* < .001) and a lower quality of life (‘my feelings’: QOLEB: *U* = 129.00, *z* = 2.22, *p* = .026, EB‐BoD: *U* = 141.00, *z* = 3.43, *p* = .001; ‘my worries and fears’: QOLEB: *U* = 141.50, *z* = 2.85, *p* = .004; EB‐BoD: *U* = 127.50, *z* = 2.77, *p* = .006). Furthermore, participants who were more burdened by their feelings were significantly less satisfied with life (SWLS: *U* = 201.00, *z* = −2.14, *p* = .033) and ranked their resources as worse (*U* = 256.50, *z* = −1.97, *p* = .049).

The accumulation of multiple stressors can be overwhelming, both for the patients and for their relatives, and may cumulate to a breakdown‐like feeling, which is linked to physical and mental exhaustion. The participants mostly refer to the following ways of dealing with it: either to acknowledge the given situation and to ‘give in and go with it’ (2788, Pos. 4), or by various ways of distraction as well as by (temporary) social isolation.

It could never be too much. We had to be ready 24/7. I got sick once for 2 days, I was on bed rest due to exhaustion. (1942, Pos. 3)

I have a breakdown and struggle for a couple of days. (1766, Pos. 4)

I breakdown and cry. I feel awful and frustrated and in pain and useless. I wish my skin would be different. I feel unlucky to have EBS as other siblings don't. I feel embarrassed when I can't walk or have to avoid certain activities. I gain weight as I can't exercise and comfort eat. (2562, Pos. 4)

I need to take a day to myself. (2015, Pos. 5)

I just want to lie on the couch and sleep the throbbing & burning foot pain away. (2789, Pos. 6)

I shut down for days, ignore everyone and cry in frustration in my room until I physically feel better. I try to distract myself mostly but sometimes it's just frustrating and upsetting to have a bad day with EB. (715, Pos. 1)

### 3.6 Resources, support and contentment

Concerning ***personal and social resources***, statistical analyses found significant results for satisfaction, helpful practices and social support. Satisfaction in relation to quality of life as measured by the ResILL‐EB Satisfaction scale was found to be lower in participants who experienced an acute challenging phase at the time point of the survey than those who did not (*U* = 178.00, *z* = −2.31, *p* = .021). With regard to helpful practices, participants who stated that their or their family member's EB was very to somewhat visible had developed more ways to deal with EB (*H*(2) = 9.37, *p* = .025), the same applies to female relatives in comparison to male (*U* = 25.00, *z* = −2.23, *p* = .026). Finally, participants who were in a relationship with a partner perceived significantly more social support as assessed by the F‐SozU (*U* = 129.00, *z* = −2.26, *p* = .024). On an individual level, some participants have found a way to deal with the fragility of their skin by paying close attention to their current condition and the state of their body. The respective knowledge and evaluation of the current skin condition is described as a skill that needs to be developed and mastered.

I genuinely think for somebody with EBS the most important part is knowing your limits. Listen to your skin. For me when I feel it starting … just stop and get off your feet asap [as soon as possible]. (2932, Pos. 5)

Learning through experience & knowing the feel of different types of blisters, when/if to burst them, how to deal with them & how long recovery will usually take. We have nicknames for the different types in my family! (2789, Pos. 7)

Relationships seem to have a beneficial impact: patients rated their current health status and quality of life significantly better when in a relationship (iscorEB: *U* = 118.50, z = 2.39, *p* = .017; QOLEB: *U* = 128.00, *z* = 2.90, *p* = .004). Moreover, they were more satisfied with life (ResILL‐EB Satisfaction scale: *U* = 18.00, *z* = −2.88, *p* = .004; SWLS: *U* = 24.00, *z* = −2.37, *p* = .018) and had more resources (ResILL‐EB Resource scale: *U* = 31.50, *z* = −2.14, *p* = .032) as well as more social support available (F‐SozU: *U* = 16.50, *z* = −2.86, *p* = .004).

**Figure 1:**
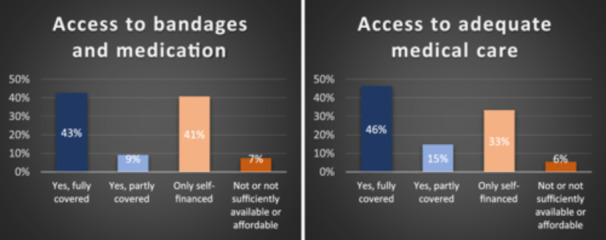
Access to bandages, medication and to adequate medical care.

In terms of financial support, more than half of the participants reported that the costs of bandages and medication were fully or partially covered and that they had full or partial access to adequate medical care. However, 41% of participants had to fully fund their own bandages and medication, and 7% reported that they did not have adequate access to bandages and medication due to a lack of funds and/or availability. Similarly, 33% of participants had only adequate access to medical care through their own finances, while 6% reported that they did not have adequate and/or affordable access (see Figure 1).

**Figure 2:**
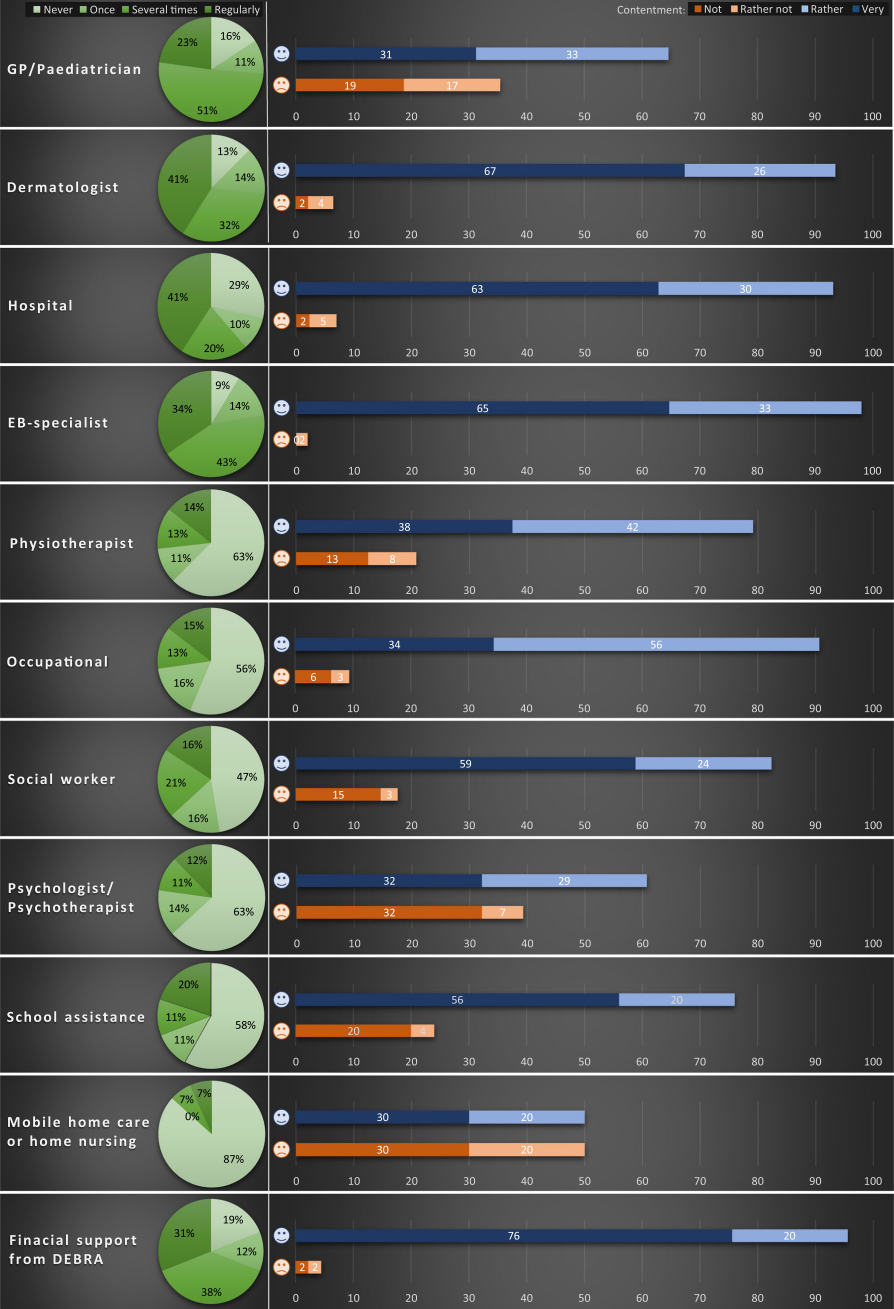
Frequency of use of medical service providers or institutions and contentment.

Furthermore, the study participants were asked if they received advice and support from the following medical service providers or institutions, and how content they were with the received advice and support of each of the service providers (see Figure 2). The medical care providers (general practitioner [GP], dermatologist, hospital and EB specialist) were used several times to regularly by more than half of the participants. Overall satisfaction ranged from 98% contentment with the EB specialist to 64% contentment with the GP. In contrast, the majority of participants never or only once used other support services (physiotherapists, occupational therapists, social workers, psychologists/psychotherapists, school assistance and mobile home care), and the contentment was lower than with the medical care providers. Especially in the area of psychological and psychotherapeutic support there seems to be a high potential for improvement. In contrast to that, financial support had been received by 81% of the participants from DEBRA Ireland at least once and 96% reported to be content with this support. Participants who reported having no financial burden had a significantly higher quality of life (for patients QOLEB: *U* = 142.50, *z* = 2.51 *p* = .012; for relatives EB‐BoD: *U* = 147.00, *z* = 3.83, *p* < .001).

To evaluate the overall support and contentment, an average score of the total received support as well as of the total contentment of those who had received support was calculated. With regard to the overall frequency, 39% of the participants indicated that they regularly received diverse advice and support and 78% were content with the services (see Figure 2).

The following statements reflect the high contentment with the received support and underlines the importance of emotional support in that respect:

Thanks to DEBRA there is a lot more support available both financial and emotional. (2198, Pos. 5)

EB nurse was very lovely and supportive. It was one of the most important things as she gave me support and understanding. (2123, Pos. 5)

The information provided by the different institutions/healthcare professionals is highly valued, too:

DEBRA Ireland support group. Important because it gave emotional support, access to clinicians and up‐to‐date research info. (2196, Pos. 6)

DEBRA Ireland are excellent in keeping us up‐to‐date with ongoing research developments and any other queries we may have from time to time. (2306, Pos. 4)

Being kept in the loop on updates in research and development. Often we only hear about new developments when we go for our appointment once a year or once every two years as it is now. (1974, Pos. 5)

However, two subgroups are less frequently receiving support and advice, are less content, and repeatedly expressed the wish for more targeted support, namely people with rather mild forms of EB and adult patients.

EB simplex being seen as a life‐impacting condition in its own right, rather than just ‘oh you have EB … but it's not dystrophic’. (2789, Pos. 5)

There has been no EB specialist in adult services in Ireland for a long time now […] This has compounded my feelings of isolation. (2831, Pos. 2)

Participants with more medical symptoms and a higher burden engaged more frequently and regularly with the diverse service providers or institutions. The frequency of the overall support correlated significantly with the iscorEB scale (*r*_*pb*_ = 0.441, *p* = .001) and the ResILL‐EB Burden scale (*r*_*pb*_ = 0.432, *p* = .001). Significant differences between the participants who received regular support and the ones with hardly any support were found on the iscorEB scale (*U* = 539.00, *z* = 2.67, *p* = .008) as well as on the ResILL‐EB Burden scale (*U* = 493.50, *z* = 2.51, *p* = .012).

Participants with more medical symptoms and fewer resources tend to seek psychological or psychotherapeutic support more frequently (iscorEB scale: *U* = 374.00, *z* = 2.37, *p* = .018 and ResILL‐EB Resources scale: *U* = 84.00, *z* = −2.99, *p* = .003).

Thereafter, the ratio of high and low service use frequency and contentment was calculated. High contentment often comes along with high frequency of service use (61.62%), whereas low contentment is equally distributed between high (21.70%) and low (21.54%) frequency of service use. Only a small percentage (12.14%) shows high contentment with low frequency of service use (Table 3).

**Table 3:** Frequency of overall support use and contentment.

	High frequency	Low frequency
High contentment	61.62	12.14
Low contentment	21.70	21.54

## 4 DISCUSSION

The aim of the current study was to evaluate burdens and helpful practices for a life with EB in Ireland.

Considering that EB is a rare disease and that in Ireland approximately 200–300 families are affected by this condition,^19^, ^20^ the total ***sample*** size of 59 study participants can be considered as highly representative. With an average age of 43.44 years, the study sample included participants from 17 to 76 years. The situation of younger children with EB was reflected through parental report by the relatives. Even though we invited all relatives to participate in our survey, the vast majority were parents of patients, leading to a higher representation of their situation with EB.

The rationale for not comparing the patient and the relatives groups, but instead analysing them jointly was mostly based on the unequal distribution of EB types as well as the severity within the two subsamples. The observed group differences can be explained by relatives being more likely to fill in the survey for their severely affected family member with EB who might not be able or have the resources to do so themselves, which might lead to some proxy report bias. Yet, this may also be a reflection of the reduced hand mobility, reported by 35% of the participants, which can occur in various EB subtypes. Future research could differentiate the identified factors by EB types.

Although there are no reported ***gender*** differences with regard to the prevalence of EB,^40^ a high percentage of women participated in the study. One possible explanation could be that women are, in general, more interested in taking part in surveys.^41^ Despite the unequal gender distribution, there were statistically significant differences with regard to burdens in the total sample. Women tend to report a higher burden due to EB. Both the higher participation and the higher burden of the female participants might be related to the fact that women mostly take over the role of the primary carer.^17^, ^42^

In our survey, the ***degree of severity*** as well as the visibility of EB were evaluated by the ratings from EB patients and relatives. Patients who were in an acute challenging phase and whose EB was very visible reported more medical symptoms, and were more burdened and less satisfied, which in turn affects their psychological well‐being and might put stress on the whole family.^17^ Although these ratings might be subjective, patients and people close to them should be considered as experts of their own or their relative's health.^43^ Our results show that those self‐reported measures are statistically relevant and related to the individually perceived burden: the higher the self‐reported degree of severity, the more medical symptoms, more burdens and fewer resources. This underlines the importance of the degree of severity as an addition to the widely used comparison of EB types, which can vary greatly within the different subtypes. Therefore, we suggest for future studies to replicate and further investigate the use of a self‐reported degree of severity of EB when assessing the current physical and psychological health status of a patient.

With regard to the ***visibility*** of EB, a negative emotional impact was reported in the qualitative as well as in the quantitative data. This reflects the earlier findings describing several stigmata associated with the visibility of EB.^12^, ^44^ Nevertheless, the invisibility was also described as a burden in the qualitative data, which can provoke a lack of understanding of the illness and its impact on the everyday life.^45^ EB patients and relatives with higher visibility, as well as female relatives, show a greater number of different helpful practices. This may be due to the higher burden on these groups.

While participants with a reduced ***mobility*** indicated to have more medical symptoms, a higher burden and less perceived satisfaction, they reported to have significantly more social support available compared to those without mobility restrictions. This could be related to the fact that help is needed in more situations, which leads to more social support.^46^, ^47^ However, reduced mobility can influence the frequency of participation in social activities and is described as a burden due to the constant necessity of planning ahead. As previously reported, reduced mobility can lead to restrictions in travelling, social events and leisure time activities and thus to a lower quality of life.^48^ The degree of severity of EB is again a contributing factor in this result. This also applies to relatives, who reported a lower quality of life due to reduced mobility of their family member with EB.

The ***financial burden*** related to EB has been previously reported and became also apparent in the current study.^18^, ^49^ Depending on the EB type, the expenses for medication, bandages and dressings are fully or partly covered by the Irish health care system.^19^ Our data show the necessity for improvement in this regard: 54% and 57% of the participants, and especially the ones with milder EB types, indicate that neither their bandages and medicines nor adequate medical care are fully covered financially. Nonetheless, additional costs may arise, for example, for special clothing for the patients, and put additional burden on many EB families. DEBRA Ireland supports families with these issues, and the high contentment with their support is reflected in the data. Nevertheless, there is still a need for improvement in this context with regard to the public health system.

In particular, the accumulation of multiple stressors related to the ***psychosocial impact*** of EB was described as overwhelming. The most common compensatory mechanisms are acceptance, adaptation to the given situation or distraction. Emotions have a high impact on the general well‐being: participants who were more burdened by their feelings were significantly less satisfied with life and ranked their resources as worse.

***Personal and social resources*** have the potential to function as a moderating factor between health‐related burdens and quality of life,^50^ which is also reflected in our data. Overall, the contentment with the support available is quite diverse. While medical support is frequently used and the contentment is high, other supports such as physiotherapy or psychotherapy are accessed less frequently and the contentment is lower. A high contentment is often associated with high frequency of service use, while low contentment is evenly distributed between high and low frequency of service use.

## 5 TRANSFER AND IMPLICATIONS

What actions should be taken, according to the participants' statements:

▪ Full publicly funded healthcare.
▪ Targeted support for:
  − Affected adults.
  − People with ‘mild’ forms of EB.
▪ Disability parking badges for EB patients (support for obtaining the badge).
▪ Psychological support for EB patients and their relatives.
▪ More awareness among the general public and healthcare professionals.

Our results showed that the vast majority of EB patients and their relatives highly appreciate the support they receive from medical service providers and institutions, especially the regular support from health care providers and by DEBRA Ireland. However, due to the financial burden of EB, the majority of patients and relatives have expressed a need ***for financial support from the state***.

Government ignorance and lack of support for patients and families. (730, Pos. 4)

Care packs should be allocated by a person's GP every month specifically for their type of EB. (2932, Pos. 4)

Free medical care and bandages plasters steroids. (2142, Pos. 2)

Two groups repeatedly expressed the wish for more ***targeted support***.

On the one hand, patients with ‘milder’ forms indicate that both the general public as well as the health care structure available are more focused on the more severe types of EB. But, as one participant puts it, all EB types should be seen as a ‘life‐impacting condition in its own right’.

Better clarification between simplex and dystrophic EB should be promoted as presently the general public can only associate with dystrophic EB. (2788, Pos. 2)

More medical and allied health professional support readily available for EB simplex, not just dystrophic. (2789, Pos. 5)

On the other hand, adults with EB refer to similar problems and to a feeling of being left out. The support already available is rather targeted towards children and the early stages of life. Therefore, affected adults bring up the need for more targeted support.

I feel as an adult there is little support … Family support seems to be more focused on the children and not parents with EB. (1899, Pos. 4)

Getting a clinic appointment for an adult is impossible, I have only been seen as an EB patient once in 8 years since I have been diagnosed. (1899, Pos. 6)

Currently in Ireland, there are no EB clinics running for adults. (2831, Pos. 3)

Many participants make quite clear statements regarding possible solutions to their struggles in regard to ***reduced mobility***. Disability parking badges could help to limit walking distances. Some participants elaborate that they would need a parking privilege only in their flare up periods (which might be a reason for difficulties obtaining the badge). From the participants' perspective, obtaining a badge is a difficult task. Targeted support could help with obtaining parking privileges and getting back some of their mobility, social participation and independence. Combined with targeted support in finding a career, this could strengthen patients' independence and sense of autonomy.

I feel it would be great if people with EB were entitled to a disabled badge to make events easier accessible. (2198, Pos. 5)

Difficulty obtaining wheelchair/disability pass for the times when my mobility is severely limited due to an EB flare so I don't have to rely on my partner to get groceries/bail out from social activities. (2789, Pos. 4)

Advising children on going forward for career choices. (1974, Pos. 2)

***Psychological support*** is central for dealing with the multifaceted psychosocial impact of EB. However, our quantitative data show that adequate psychological counselling is not so easy to find: Whereas 61% of our participants are very satisfied or satisfied with their psychological care, 39% indicate to be rather not or not satisfied. Nevertheless, both the patients as well as their relatives articulate the wish for professional psychological or psychotherapeutic support, ideally linked to the medical care. Whether this may take place on a one‐time or regular basis, in specialised facilities or in private practice, important in this matter are destigmatisation, accessibility and affordability.

Psychological help for all the family from birth. (1765, Pos. 3)

More support for grandparents and extended family that are heavily involved in care. Psychological support for EB patient is non‐existent, they need more help. (2123, Pos. 3)

Psychological and mental health therapy for parents of children with a terminal diagnosis. Our children were completely cared for by hospital and carers, but us as their primary carers did not find as much support, particularly in the dealing with our anxieties and general mental health. (1766, Pos. 3)

Clinical psychology to provide 1:1 psychotherapy should be freely available and linked to the treating team (if I had one!) so that the person providing psychotherapy can liaise with the medical team in relation to management and prognosis. (2831, Pos. 3)

Due to the complexity of EB, psychological guidance for people with EB and their relatives needs specific training. Further research and practical recommendations on how to strengthen personal resources in a context of EB would be helpful to better support EB patients and their relatives in Ireland.

Several participants expressed their wish for more ***awareness*** among the general public and by healthcare professionals. For the general public, one could consider public awareness campaigns like the successful poster campaigns in Austria^51^ or the ALS ice bucket challenge.^52^ For daily interactions with people who have no or not sufficient knowledge of EB, it could be helpful to provide the EB patients and their families with structured communication skills on how to explain EB.^53^ For more awareness about EB in the medical field, specialised EB trainings should be offered to healthcare professionals.

Lack of understanding of EB by family and general public. Important as it makes it isolating. (2350, Pos. 3)

Education programmes for family members. (2417, Pos. 7)

Lack of treatment and lack of specialised clinical care because it is frustrating for me and my family. (2196, Pos. 4)

## 6 CONCLUSION AND OUTLOOK

The findings of the current survey highlight the importance of considering both the burdens associated with EB and the helpful practices in dealing with this rare disease to improve the quality of life of EB patients and their family members. Our findings can be used as a basis for the development of targeted psychosocial intervention programmes for EB patients as well as for their relatives in Ireland, to strengthen existing resources and to develop strategies for a life with EB.

## AUTHOR CONTRIBUTIONS

**Gudrun Salamon**: Conceptualisation; methodology; investigation; supervision; funding acquisition; project administration; writing—original draft; writing—review and editing; data curation; formal analysis; visualisation; resources. **Ursula Field‐Werners**: Data curation; formal analysis; writing—original draft. **Sophie Strobl**: Data curation; investigation; visualisation; writing—original draft; writing—review and editing; formal analysis. **Vinzenz Hübl**: Formal analysis; writing—review and editing. **Anja Diem**: Writing—review and editing; resources; supervision.

## CONFLICT OF INTEREST STATEMENT

The authors declare no conflict of interest.

## ETHICS STATEMENT

This study was performed in line with the principles of the Declaration of Helsinki. Ethical approval was granted by both the Medical and the Psychological Ethics Committees of the Sigmund Freud University Vienna (LBVCR72KAUBQ6188306).

## Supporting information

Supporting information.

## Data Availability

The data are not publicly available due to privacy or ethical restrictions. Despite the quantitative study design, data publication would violate ethical principles: given the rarity of the disease, the large number of subtypes and the strong interconnectedness within the patient community, already a combination of basic demographic data such as country, age and EB subtype could lead to recognition of individuals. Thus, even anonymisation would not allow sufficient de‐identification of the data as required by ethical and data protection regulations.

## Footnotes

* For readers who are not familiar with the Irish healthcare system and its services for people with EB, we recommend the lecture of Kearney et al.^23^ and Gowran et al.^24^ For a description of the patient organisation DEBRA Ireland and their services, please visit https://www.debra.ie.
