# Supplementary material for: The psychosocial impact of a chronic disease in Ireland: Burdens and helpful practices for a life with epidermolysis bullosa
Source: Health Expect. 2024 Jun 21;27(3):e14088. doi: 10.1111/hex.14088 (PMC11192844; doi:10.1111/hex.14088)
Supplement: Supplementary file 1 — Supporting information. [file HEX-27-e14088-s001.pdf]

The Psychosocial Impact of a Chronic Disease in Ireland: Burdens and Helpful Practices for a Life with Epidermolysis Bullosa

Gudrun Salamon, Ursula Field-Werners, Sophie Strobl, Vinzenz Hübl, Anja Diem

Additional Material

Table A1. Distribution of degree of severity by EB type

	Mild	Moderate	Severe
**EB simplex** (EBS)	19 (79.2%)	17 (77.3%)	2 (15.4%)
**Dystrophic EB** (DEB)	5 (20.8%)	5 (22.7%)	8 (61.5%)
**Junctional EB** (JEB)	0 (0%)	0 (0%)	3 (23.1%)

Table A2. Group comparison matrix of all categories' medians

	Current health ↓	Quality of life ↓		Burdens ↑	Satisfaction with EB ↑	Resources ↑	Helpful strategies ↑	Resilience ↑	Social support ↑	Overall satisfaction ↑
	iscorEB	For patients: QOLEB	For relatives: EB-BoD	ResILL-EB Burden scale	ResILL-EB Satisfaction scale	ResILL-EB Resources scale	ResILL-EB Helpful strategies	BRS	F-SozU	SWLS
**Gender**	*No significant differences*	*No significant differences*	*p*=.012	*p*=.042	*No significant differences*	*No significant differences*	*p*=.026*2	*No significant differences*	*No significant differences*	*No significant differences*
Female			MD=2.44	MD=3.40			MD=1.71			
Male			MD=0.50	MD=1.63			MD=1.42			
**Severity**	*p*<.001	*No significant differences*	*p*=.007	*p*<.001	*No significant differences*	*p*=.044	*No significant differences*	*No significant differences*	*No significant differences*	*No significant differences*
Mild	MD=1.27		MD=1.00	MD=1.95		MD=2.20				
Moderate	MD=1.87		MD=1.87	MD=2.80		MD=2.00				
Severe	MD=4.40		MD=4.40	MD=7.10		MD=1.80				
**Visibility**	*p*=.043	*No significant differences*	*No significant differences*	*p*=.046	*No significant differences*	*No significant differences*	*p*=.025	*No significant differences*	*No significant differences*	*No significant differences*
Not	MD=1.47			MD=2.48			MD=1.59			
Hardly				MD=1.95			MD=1.82			
Somewhat	MD=1.87			MD=2.98			MD=1.88			
Very				MD=7.08			MD=1.65			
**Acute EB phase**	*p*=.027	*p*=.019	*No significant differences*	*p*=.038*1	*p*=.021	*No significant differences*	*No significant differences*	*No significant differences*	*No significant differences*	*No significant differences*
Yes	MD=2.07	MD=1.88		MD=4.50	MD=4.56					
No	MD=1.47	MD=1.59		MD=1.83	MD=6.67					
**Limited mobility**	*p*=.046	*No significant differences*	*p*=.005	*p*=.026	*p*=.010	*p*=.015	*No significant differences*	*No significant differences*	*No significant differences*	*p*=.001
Yes	MD=2.00		MD=3.06	MD=3.55	MD=5.44	MD=1.90				MD=3.80
no	MD=1.40		MD=0.86	MD=1.83	MD=6.89	MD=2.15				MD=5.80
**Overall support**	*p*=.008	*No significant differences*	*No significant differences*	*p*=.012	*No significant differences*	*No significant differences*	*No significant differences*	*No significant differences*	*No significant differences*	*No significant differences*
Frequently	MD=2.40			MD=6.70						
No support	MD=1.52			MD=2.58						
**Psychologic/ therapeutic support**	*p*=.018	*No significant differences*	*p*=.002	*No significant differences*	*No significant differences*	*p*=.003	*No significant differences*	*No significant differences*	*No significant differences*	*No significant differences*
Frequently	MD=2.40		MD=3.44			MD=1.80				
Not/once	MD= 1,47		MD=1.28			MD=2.10				
**Financial burden**	*p*=.001	*p*=.012	*p*<.001	*No significant differences*	*p*=.031	*No significant differences*	*No significant differences*	*No significant differences*	*No significant differences*	*p*=.015
Burdened	MD=1.40	MD=1.76	MD=3.43		MD=5.44					MD=4.00
Not/rather not	MD=2.40	MD=1.56	MD=0.86		MD=6.78					MD=5.60
**Financial satisfaction**	*No significant differences*	*p*=.017	*No significant differences*	*No significant differences*	*No significant differences*	*No significant differences*	*No significant differences*	*No significant differences*	*No significant differences*	*p*=.004
Satisfied		MD=1.59								MD=5.70
Not/rather not		MD=1.76								MD=3.80
**Mood**	*No significant differences*	*p*=.001	*p*=.015	*p*=.002	*p*=.023	*p*<.001	*No significant differences*	*p*=.007	*No significant differences*	*p*=.001
Happy		MD=1.65	MD=1.56	MD=2.51	MD=6.50	MD=2.16		MD=3.33		MD=5.60
Unhappy		MD=2.12	MD=3.43	MD=6.00	MD=4.17	MD=1.75		MD=2.67		MD=3.00
**Burden due to feelings**	*p*<.001	*p*=.026	*p*=.001	*No significant differences*	*No significant differences*	*p*=.049	*No significant differences*	*No significant differences*	*No significant differences*	*p*=.033
Burdened	MD=2.53	MD=1.82	MD=3.22			MD=1.90				MD=3.80
Not/hardly	MD=1.33	MD=1.65	MD=0.81			MD=2.10				MD=5.40
**Burden due to worries and fears**	*p*<.001	*p*=.004	*p*=.006	*No significant differences*	*No significant differences*	*No significant differences*	*No significant differences*	*No significant differences*	*No significant differences*	*No significant differences*
Burdened	MD=2.40	MD=1.94	MD=3.06							
Not/hardly	MD=1.33	MD=1.59	MD=0.94							
**Relationship**	*p*=.017*1	*p*=.004	*No significant differences*	*No significant differences*	*p*=.004*1	*p*=.032*1	*No significant differences*	*No significant differences*	*p*=.024	*p*=.018*1
Yes	MD=1.33	MD=1.65			MD=6.61	MD=2.16			MD=3.83	MD=5.60
No	MD=2.13	MD=1.94			MD=3.44	MD=1.80			MD=3.00	MD=3.80

**1 significant differences in patients only, *2 significant differences in relatives only*

↑= Higher values indicate higher expression in the targeted construct; ↓= Lower values indicate higher expression in the targeted construct
